# Down-regulation of ERAP1 mRNA expression in non-small cell lung cancer

**DOI:** 10.1186/s12885-023-10785-7

**Published:** 2023-04-26

**Authors:** Marta Wagner, Maciej Sobczyński, Monika Jasek, Konrad Pawełczyk, Irena Porębska, Piotr Kuśnierczyk, Andrzej Wiśniewski

**Affiliations:** 1 Laboratory of Genetics and Epigenetics of Human Diseases, Hirszfeld Institute of Immunology and Experimental Therapy, Polish Academy of Sciences, Wrocław, Poland; 2 Laboratory of Molecular Neurobiology, Nencki Institute of Experimental Biology of the Polish Academy of Sciences, Warsaw, Poland; 3 Department of Thoracic Surgery, Lower Silesian Centre of Oncology, Pulmonology and Haematology, Wrocław, Poland; 4 Department of Pulmonology and Lung Oncology, Wrocław Medical University, Wrocław, Poland; 5 Laboratory of Immunogenetics and Tissue Immunology, Hirszfeld Institute of Immunology and Experimental Therapy, Polish Academy of Sciences, Wrocław, Poland

**Keywords:** Non-small cell lung cancer, *ERAP1* mRNA expression, rs26653, eQTL, Genetics

## Abstract

**Background:**

ERAP1 is a major aminopeptidase that serves as an editor of the peptide repertoire by trimming N-terminal residues of antigenic peptides, creating a pool of peptides with the optimal length for MHC-I binding. As an important component of the antigen processing and presenting machinery – APM, ERAP1 is frequently down-regulated in many cancers. Since ERAP1 expression has not yet been thoroughly investigated in non-small cell lung cancer (NSCLC), we decided to analyze *ERAP1* mRNA levels in tissues collected from NSCLC patients.

**Methods:**

Using real-time qPCR, we evaluated *ERAP1* mRNA expression in samples of tumor and adjacent non-tumor tissue (serving as control tissue) from 61 NSCLC patients.

**Results:**

We observed a significantly lower level of *ERAP1* mRNA expression in tumor tissue (Med_Tumor_ = 0.75) in comparison to non-tumor tissue (Med_Non-tumor_ = 1.1), *p* = 0.008. One of the five tested polymorphisms, namely rs26653, turned out to be significantly associated with *ERAP1* expression in non-tumor tissue (difference [d] = 0.59 CI95% (0.14;1.05), *p* = 0.0086), but not in tumor tissue.

The levels of *ERAP1* mRNA expression did not affect the overall survival of NSCLC patients, either in the case of the tumor (*p* = 0.788) or in non-tumor (*p* = 0.298) tissue.

We did not detect any association between mRNA *ERAP1* expression level in normal tissue and: (i) age at diagnosis (*p* = 0.8386), (ii) patient’s sex (*p* = 0.3616), (iii) histological type of cancer (*p* = 0.7580) and (iv) clinical stage of NSCLC (*p* = 0.7549). Furthermore, in the case of tumor tissue none of the abovementioned clinical parameters were associated with *ERAP1* expression (*p* = 0.76).

**Conclusion:**

Down-regulation of *ERAP1* mRNA observed in NSCLC tissue may be related to tumor immune evasion strategy. The rs26653 polymorphism can be considered an expression quantitative trait *locus* (eQTL) associated with ERAP1 expression in normal lung tissue.

**Supplementary Information:**

The online version contains supplementary material available at 10.1186/s12885-023-10785-7.

## Introduction

To avoid immune-mediated recognition and elimination, tumors adopt different strategies, including the loss of their antigenicity through the reduced expression of major histocompatibility class I complexes (MHC-I) or dysregulation of the antigen processing machinery – APM components that are responsible together for the production and presentation of tumor antigens [1]. Defects and/or alterations in antigen processing and the presentation pathway significantly change both the peptide supply and the presented peptide repertoire at the tumor cell surface. Occurring either independently or cumulatively, the defects within the APM ultimately result in the loss of peptides-MHC-I complexes (pMHC-I) or a change in the repertoire of presented peptides [2]. The endoplasmic reticulum aminopeptidase 1—ERAP1 plays a key role in generating the optimal length of peptides for MHC class I binding [3]. ERAP1 trims preferentially peptides longer than 9-mers and becomes virtually inactive with 8-mers and shorter peptides [4].

The expression of ERAP1 is frequently altered in tumors, when compared to normal, non-cancerous tissues [5], but how this affects tumor growth and the activation of anti-tumor immune responses is still not understood well [3]. Alterations in the protein expression of ERAP1 have been detected in various solid tumors, including carcinomas of the breast, ovary, liver, lung, colon and pancreas [6]. In esophageal carcinoma lesions, the expression of ERAP1 was lost or down-regulated in 20 and 28% of cases, respectively, and significantly associated with the depth of tumor invasion [7]. In patients affected by triple-negative breast cancer, low expression of ERAP1 has been associated with poor clinical outcomes [8]. In another study, in cervical carcinoma patients, partial ERAP1 loss was significantly associated with reduced 5-year overall survival (OS), disease free survival, and the presence of lymph node metastases [9].

Since ERAP1 expression levels have not yet been thoroughly investigated in non-small cell lung cancer (NSCLC), we decided to analyze *ERAP1* mRNA levels in tissues collected from NSCLC patients. Additionally, we checked whether some *ERAP1* SNPs, which were found to be associated with NSCLC predisposition (as shown in our last report [10]), correlated with *ERAP1* mRNA levels in tumor and adjacent non-tumor tissue. We also attempted to determine whether *ERAP1* mRNA levels measured in the tumor are associated with some clinical parameters.

## Materials and methods

### Study subjects

In total, 72 newly diagnosed patients with pathologically documented NSCLC were recruited in the Department of Thoracic Surgery, Lower Silesian Centre of Oncology, Pulmonology and Haematology in Wrocław (Poland). All of our patients were of Polish origin. The histological type of lung cancer and pathologic stages were determined according to the World Health Organization (WHO 2015) classification and the International System for Staging Lung Cancer [11], respectively. Detailed characteristics of the patients are shown in Table 1. Patients with a history of primary cancer other than lung cancer were excluded from the study. Before deciding on surgical treatment, all the patients had a chest radiograph, chest computed tomography (CT) scan, bronchoscopy, abdominal ultrasound and CT/MRI of the central nervous system, in case of neurological symptoms. Positron emission tomography (PET-CT) was performed in any cases of doubt, before invasive diagnosis of mediastinal lymph nodes and in any cases of the diagnosis of mediastinal and peripheral changes due to suspected metastases. In cases where there was suspicion of metastases to the mediastinal lymph nodes during preoperative imaging (> 1 cm diameter in a short axis), an endobronchial ultrasound biopsy (EBUS) or videomediastinoscopy was performed. Patients with N2 disease were excluded from surgical treatment. Surgical treatment in the studied group of patients included anatomical resection of the lung parenchyma (lobectomy, bilobectomy, pneumonectomy) and systematic mediastinal lymph node dissection with "en bloc" resection of the right paratracheal 2R and 4R nodes. Nodes of 4L, 5, 6, 7, 8, 9, 10 and 11 groups were removed separately. Intrapulmonary nodes of group 12 were removed together with a lobe, then carefully resected and evaluated by a histopathologist. Pathological staging was evaluated based on the current edition of the TNM, all the stages were revised from the pathology reports and the Polish National Lung Cancer Registry. In the case of a confirmed pN1 or pN2, stage patients were qualified for adjuvant chemotherapy or radio/chemotherapy.

**Table 1:** Characteristics of NSCLC patients

**Age**	**n**	**Min**	**Q1**^**a**^	**Median**	**Q3**^**a**^	**Max**	Variability^1^
Men	36	52	61	66.5	71	81	7
Women	36	49	60	64	68	81	5
All	72	49	60	65	70	81	6
**Histological type**	**LUSC**	**LUAD**	**Other**	**% LUSC**	**% LUAD**	**% Other**	Variability^2^
Men	16	13	7	*44*	*36*	*19*	0.95
Women	6	26	4	*17*	*72*	*11*	0.71
All	22	39	11	*31*	*54*	*15*	0.89
**TNM**^c^	**T1**	**T2**	**T3**	**T4**	**N0**	**N1**	**N2**
Men	9	13	11	3	24	12	0
Women	15	15	6	0	27	4	5
All	24	28	17	3	51	16	5
% All	*33*	*39*	*24*	*4*	*71*	*22*	*7*
**Tumor stage**	**I**	**II**	**III**	**% I**	**% II**	**% III**	Variability^2^
Men	16	13	7	*44*	*36*	*19*	0.95
Women	24	6	6	*67*	*17*	*17*	0.79
All	40	19	13	*56*	*26*	*18*	0.9
**Smoking**	**0 p-y**	**5.5**	**15.5**	**25.5**	**35.5**	**45.5**	Variability^2^
Men	0	1	4	6	17	8	0.74
Women	4	1	5	11	13	2	0.84
All	4	2	9	17	30	10	0.84
% All	*5*	*3*	*12*	*24*	*42*	*14*	-
**OS**	**All patients**			**Death patients**			Variability^1b^
	**Min**	**Median**	**Max**	**Min**	**Median**	**Max**	
Men	0.7	23.0	35.0	0.7	10.9	21.3	10.7
Women	5.2	25.8	35.4	5.2	9.2	22.2	7.5
All	0.7	24.8	35.4	0.7	9.2	22.2	9.1

^a^1st and 3rd quartile

^b^all patients

^c^all patients M0

^1^${S}_{n}$-typical difference between two randomly chosen patients

^2^Normalized Shannon’s entropy – measure between [0,1], where entropy = 1 is maximal variability; For more information see *Statistical methods*

*LUSC* Lung squamous cell carcinoma, *LUAD* Lung adenocarcinoma, *p-y* Pack-years, *OS* Overall survival

Overall survival was assessed from the date of cancer diagnosis until death from any cause or until two years when data collection was completed.

For each patient, information about their smoking history was available. Only four subjects were never-smokers (0 pack-years). The rest of the patients were smokers currently or in the past (quitting at least one year before diagnosis). As can be seen from Table 1, male patients generally smoked more cigarettes, were diagnosed at more advanced disease stages, and survived shorter in comparison to female patients.

### RNA Isolation And Quantification of *ERAP1* mRNA Levels

#### RNA isolation and cDNA synthesis

Total RNA was isolated from 1) formalin-fixed paraffin-embedded (FFPE) tumor tissues as well as adjacent non-tumor tissues from 39 NSCLC patients (A) and 2) tumor and adjacent non-tumor tissues stabilized in AllProtect Tissue Reagent from 22 individuals with NSCLC (B).

A. Extraction from FFPE tissues was performed with Quick-DNA/RNA™ FFPE Kit (Zymo Research) following the manufacturer’s protocol except for the deparaffinization procedure performed with Qiagen Deparaffinization Solution. RNA concentration and purity were assessed using the NanoDrop 2000 spectrophotometer (Thermo Scientific). RNA integrity was analyzed by gel electrophoresis. cDNA was synthesized from 1 µg of total RNA using the iScript cDNA Synthesis Kit for RT-qPCR (Bio-Rad) following the manufacturer’s instruction.
B. Total RNA from tissue stabilized in AllProtect Tissue Reagent was isolated using AllPrep DNA/RNA/miRNA Universal Kit (Qiagen) according to the manufacturer’s instruction with some exceptions. Namely, 1) lysate was treated with QIAshredder (Qiagen) homogenizer to filter out insoluble debris and reduce viscosity, and 2) DNase treatment was extended to 45 min. RNA concentration and purity were assessed using NanoDrop 2000 spectrophotometer (Thermo Scientific). RNA integrity was also analyzed by gel electrophoresis. Commercially available Human Lung Total RNA (Thermo Fisher Scientific) was used to standardize the experiments. cDNA was synthesized from 2-6 µg of total RNA (depending on the isolation yield) using iScript Advanced cDNA Synthesis Kit for RT-qPCR (Bio-Rad) following the manufacturer’s protocol. The generated cDNA samples were diluted to the equivalent of 50 ng/µl. Negative controls with no reverse transcriptase (RT-) were also prepared to exclude contamination with genomic DNA.

#### qPCR

To investigate *ERAP1* mRNA expression, qPCR was performed using TaqMan Gene Expression Assays in LightCycler480 II system with a 96-well plate format. GAPDH and ACTB were used as reference genes. The following TaqMan Gene Expression Assays were used in the study: ERAP1 (Hs00429970_m1), GAPDH (Hs03929097_g1), and ACTB (Hs01060665_g1).

To determine the amplification efficiencies (E) for particular assays the relative standard curves were generated using a five-point two-fold serial dilution of the cDNA. The corresponding efficiencies (E) were calculated according to the following formula: $E={(10}^{\frac{-1}{slope}}-1)\times 100$. Calculated efficiencies were taken into account in further calculations (please see below).

Each qPCR reaction with a final volume of 15 μl included 1 × TaqMan Gene Expression Master Mix (7.5 μl), 1 × TaqMan Gene Expression Assay (0.75 μl), 2 μl of cDNA and 4.75 μl of DNase/RNase-free water. qPCR reaction conditions were as follows: 95˚C for 10 min and 45 cycles at 95˚C for 15 s and 60˚C for 1 min. All reactions were run in triplicates. Negative template control was run on each plate. Regarding the approach of the run layout, we selected the sample maximization method. However, since not all of the samples could be analyzed in the same run, an inter-run calibrator (the same sample) was included in each plate to correct for possible run-to-run variation. The variation between runs was acceptably low; therefore, the calibrator factor was not included in the calculations. Thus, normalized relative quantities (NRQ) were calculated from the following equation:

$$NRQ=\frac{{E}_{goi}^{\Delta Cq}}{\sqrt[n]{\prod {E}_{ref, i}^{\Delta Cq}}}$$

where $goi$ – gene of interest, $ref$ – reference gene, $E$—amplification efficiency, $\Delta Cq$—the difference between a reference $Cq$ value (the average $Cq$ across all samples) and the $Cq$ value for the sample of interest.

### Correlation with available genotypic data

For the next stage, we wanted to find out whether the level of *ERAP1* mRNA expression is correlated with genotypes at five polymorphic sites in *ERAP1*: rs26653, rs26618, rs2287987, rs30187, rs27044. These SNPs were selected and genotyped in our earlier study concerning NSCLC [10]. Unfortunately, genotypic data were available only for the 35 patients from the present study. All tested polymorphisms were in Hardy–Weinberg equilibrium.

### Statistical analysis

Data characterizing NSCLC patients were described with median, first and third quartiles (Q1 and Q3, respectively) as well as minimal and maximal values. The ${S}_{n}$ statistic was computed as the measure of variability: ${S}_{n}=med\left\{med\left|{x}_{i}-{x}_{j}\right|i,j=1,..n\right\}$. ${S}_{n}$ is the typical distance between two randomly selected individuals and can be used as a measure of variability instead of standard deviation when the median is used instead of the arithmetic mean [12]. In the case of ordinal and nominal variables with $k=\mathrm{1,2},\dots ,K$ categories (levels) standardized Shannon’s entropy ${E}_{S }$ was used as the measure of variability, where ${E}_{S}=-\frac{1}{{\mathrm{log}}_{2}K}{\sum }_{k}\widehat{f}\left(k\right){\mathrm{log}}_{2}\widehat{f}\left(k\right)\in \left[\mathrm{0,1}\right]$ and ${E}_{S}=1$ is maximal possible variability when every level $k$ occurs with the same frequency $\widehat{f}\left(k\right)$ in the sample and ${E}_{S}=0$ in case when only one level of an analyzed variable is observed in the sample.

Levels of *ERAP1* mRNA expression in tumor and non-tumor tissues (Tables 2 and  3) were described as mentioned above. The location parameter was estimated with the Hodges – Lehmann estimator with percentile-based 95%-confidence intervals (95%CI). CIs for it was estimated with bootstrap sampling ($B=10 000$ samples). A test for the difference between the average expression in tumor and non-tumor tissues (Table 2) was performed on bootstrap estimated distribution. Linear regression was used to test the relationships between *ERAP1* mRNA expression and clinical and demographical factors with standard errors estimated based on residual-based bootstrap sampling. Survival was analyzed with proportional hazards models.

**Table 2:** *ERAP1* mRNA expression in tumor and non-tumor tissue among NSCLC patients

Tissue	Min	Q1^a^	Median^b^	CI95% for median^c^		Q3^a^	Max	Variability^d^
Non-tumor	0.03	0.72	1.10	0.94	1.29	1.50	4.23	0.64
Tumor	0.02	0.34	0.75	0.60	0.91	1.09	3.56	0.49
Difference	− 3.95	− 0.80	− 0.30	− 0.52	− 0.09	0.15	1.78	0.68
	*p* = 0.008							

^a^1^st^ and 3^rd^ quartile

^b^Hodges – Lehmann estimator

^c^Estimated with bootstrap sampling

^d^$Sn$ statistic – typical difference between two randomly chosen persons

**Table 3:** Levels of *ERAP1* mRNA expression in tumor and non-tumor tissue according to sex, histological type of NSCLC and tumor stage

Variable	Group	Non-tumor				Tumor			
		**Median**^a^	**CI95%**^b^		**Variability**^c^	**Median**^a^	**CI95**^b^		**Variability**^c^
Sex	Men	1.21	0.95	1.46	0.62	0.71	0.51	0.94	0.47
	Women	1.00	0.76	1.27	0.54	0.80	0.58	1.04	0.52
Histological type of NSCLC	LUSC	1.15	0.75	1.55	0.80	0.70	0.47	0.96	0.39
	LUAD	1.08	0.88	1.28	0.48	0.82	0.56	1.11	0.62
	Other	1.20	0.66	2.12	0.72	0.70	0.37	1.03	0.51
Tumor stage	I	1.05	0.84	1.27	0.54	0.89	0.67	1.11	0.48
	II	1.44	0.97	1.98	0.75	0.59	0.36	0.84	0.31
	III	0.89	0.48	1.30	0.60	0.64	0.26	1.20	0.35
TNM	T1	0.98	0.68	1.31	0.56	0.90	0.59	1.26	0.61
	T2	1.09	0.86	1.37	0.46	0.76	0.54	0.99	0.43
	T3-T4	1.17	0.95	1.42	0.56	0.62	0.37	0.92	0.40
	N0	1.12	0.90	1.34	0.62	0.78	0.62	0.96	0.47
	N1	1.33	0.93	1.82	0.32	0.60	0.25	1.19	0.24
	N2	0.59	0.10	1.12	0.35	0.82	0.24	1.43	0.52

^a^Hodges – Lehmann estimator

^b^Estimated with bootstrap sampling

^c^$Sn$ statistic – typical difference between two randomly chosen persons

*LUSC* Lung squamous cell carcinoma, *LUAD* Lung adenocarcinoma

Genotype-dependent *ERAP1* mRNA expression levels were tested and estimated with the methods described above. Deviation from Hardy–Weinberg Equilibrium (HWE) was measured as $f=\frac{{p}_{aa}-{p}_{a}^{2}}{{p}_{a}(1-{p}_{a})}$ where ${p}_{a}$ and ${p}_{aa}$ are allele $a$ and genotype $aa$ frequencies. Negative and positive $f$ values correspond to a deficiency and excess of homozygotes, respectively, and $f = 0$ in the case of HWE. The distribution for null H0: *locus is in HWE* was estimated exactly via Monte Carlo simulation.

## Results

**Fig. 1:**
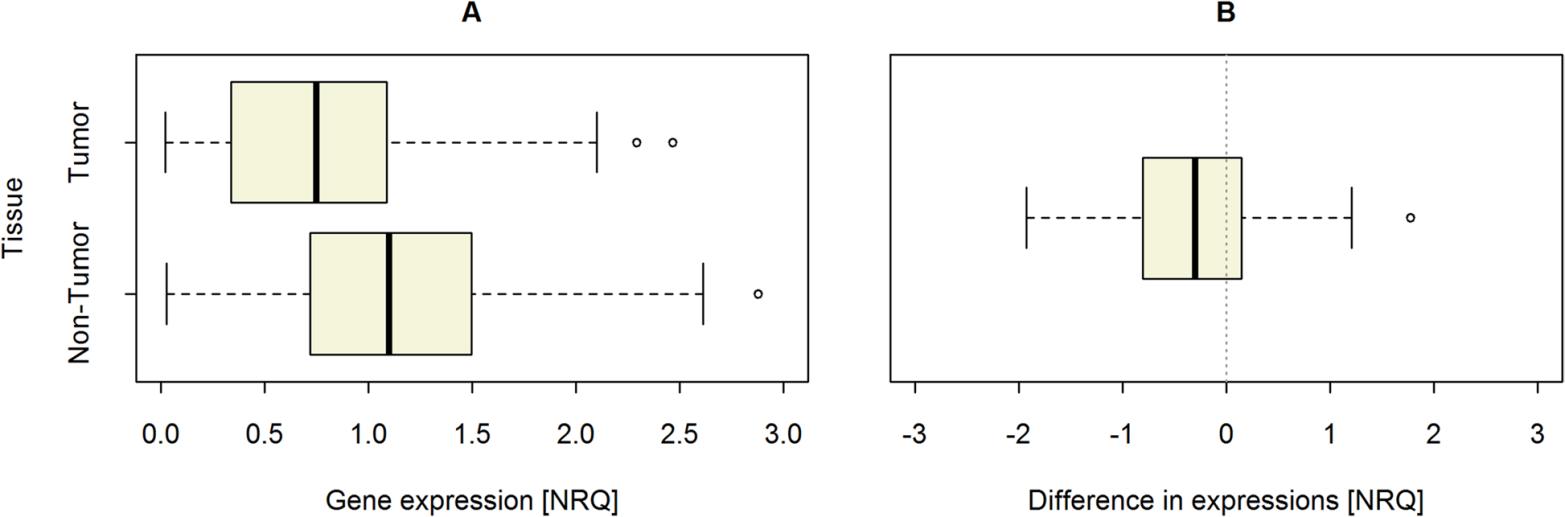
*ERAP1* mRNA expression in tumor and non-tumor tissue (**A**) as well as a difference between two types of tissue in NSCLC patients (**B**)

Our study revealed that the average level of *ERAP1* mRNA expression in non-tumor tissue was significantly higher than in tumor tissue of the same patient, as shown in Table 2. In detail, median mRNA expression level in non-tumor tissue was Med_Non-tumor_ = 1.1, whereas in tumor tissue – Med_Tumor_ = 0.75; *p* = 0.008. This difference is also depicted in Fig. 1. The average difference between non-tumor and tumor tissues reached Med_d_ =  − 0.3, with the average difference between two randomly chosen patients S_*n* =_ 0.68 (Table 2).

Table 3 presents the levels of *ERAP1* mRNA expression according to sex, histological type of NSCLC and tumor stage. Due to the very low correlation of expression levels in tumor and non-tumor tissue from the same patient (${r}_{Pearson}=0.22$), we considered them separately. We did not detect any association between mRNA *ERAP1* expression level in normal tissue and: (i) age at diagnosis (*p* = 0.8386), (ii) patient’s sex (*p* = 0.3616), (iii) histological type of cancer (*p* = 0.7580) and (iv) clinical stage of NSCLC (*p* = 0.7549). Furthermore, in tumor tissue none of the abovementioned clinical parameters were associated with *ERAP1* expression (F_5;55_ = 0.52, *p* = 0.76). Moreover, none of the variables mentioned above were related to the difference in *ERAP1* mRNA expression between tumor and non-tumor tissue from a given patient.

The level of *ERAP1* mRNA expression did not affect the overall survival of patients with NSCLC, either in the case of the tumor (*p* = 0.788) or in non-tumor (*p* = 0.298) tissue. These results were adjusted to the smoking status, age of diagnosis, sex, staging, and histological type of NSCLC. Table 4 shows the average overall survival in the four groups (I-IV) divided according to the level of *ERAP1* mRNA expression in tumor and non-tumor tissues. No significant differences in OS were observed between the groups. Moreover, we can conclude that the expression levels were independent, as the number of patients in each group is similar (expected number: 31/4 = 7.75), (Table 4).

**Table 4:** Average overall survival according to the level of *ERAP1* mRNA expression in tumor and non-tumor tissues (for living patients, *n* = 31)

		***ERAP1* mRNA expression in non-tumor tissues**	
		Low^a^	High^b^
***ERAP1* mRNA expression in tumor tissues**	Low^a^	(I) Median OS = 29.5 (*n* = 10)	(III) Median OS = 27.0 (*n* = 6)
	High^b^	(II) Median OS = 31.3 (*n* = 6)	(IV) Median OS = 25.8 (*n* = 9)

Overall survival data [months] were available only for 39 patients (31 living patients and 8 died)

^a^Low expression (≤ Median expression)

^b^High expression (> Median expression)

**Fig. 2:**
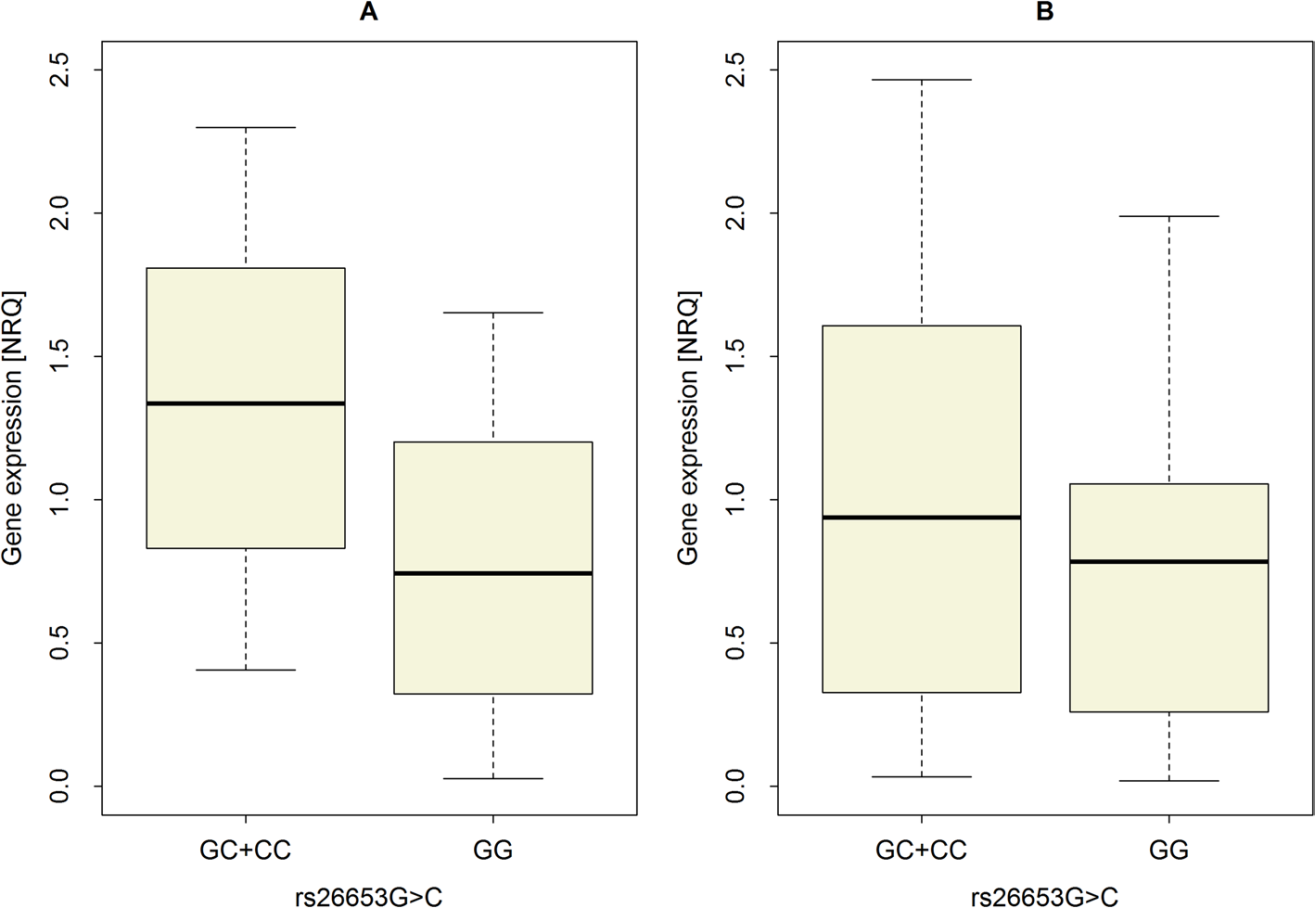
*ERAP1* mRNA expression in non-tumor (**A**) and tumor (**B**) tissue depending on genotype in rs26653 G > C *locus*

We also investigated the possible impact of several *ERAP1* variants on mRNA expression levels in tumor and non-tumor tissue. Only polymorphism rs26653 turned out to be significantly associated with *ERAP1* expression, but exclusively in non-tumor tissue (difference d = 0.59 CI95% [0.14;1.05] *p* = 0.0086), (Table 5). Since the frequency of the minor allele in the case of rs26653 polymorphism is low (among 35 patients analyzed here, there was only one homozygous CC individual), we pooled the genotypes CC and GC in one group and compared the minor allele carriers (CC + GC) to the group of common homozygotes (GG). The median *ERAP1* expression value in this group was 1.33, CI95% [0.94;1.69], whereas for GG genotypes median expression value was about twofold lower and achieved the level of 0.74 with CI95% [0.47; 1.00]. For comparison, in tumor tissue the difference between both groups of genotypes was small and achieved d = 0.09, CI95% [− 0.40;0.68], *p* = 0.72 (Table 5). Expression of *ERAP1* in tumor and non-tumor tissue according to genotype in *locus* rs26653 was also shown in Fig. 2. Other tested polymorphisms did not correlate with *ERAP1* mRNA levels either in cancer or in normal tissue (Table 5).

**Table 5:** Genotype-dependent *ERAP1* mRNA expression levels as well as HWE test and $f$ measure of *locus* deviation from Hardy–Weinberg equality

**SNP**	**ERAP1 expression**	**Genotype**	**CC**	**GC**	**GG**
rs26653 G > C		n	1	15	19
		%	*2.9*	*42.9*	*54.3*
	Non-tumor	Median^a^	1.33^d^		0.74
		CI95%^b^	0.94; 1.69		0.47; 1.00
		Difference	d = 0.59 CI95% (0.14; 1.05), *p* = 0.0086		
	Tumor	Median^a^	0.94^d^		0.78
		CI95%^b^	0.46; 1.56		0.49; 1.07
		Difference	d = 0.09 CI95% (− 0.40; 0.68), *p* = 0.7248		
	**HWE test**^c^	$\chi 2$= 0.96, *p* = 0.32;$f$= − 0.17, CI95% (− 0.4; 0.12)			
rs26618 T > C	**ERAP1 expression**	**Genotype**	**CC**	**TC**	**TT**
		n	2	13	20
		%	*5.7*	*37.1*	*57.1*
	Non-tumor	Median	1.11		0.90
		CI95%	0.75; 1.46		0.57; 1.23
		Difference	d = 0.21 CI95% (− 0.28; 0.66), *p* = 0.3874		
	Tumor	Median	0.82		0.84
		CI95%	0.41; 1.46		0.52; 1.18
		Difference	d = − 0.03 CI95% (− 0.53; 0.50), *p* = 0.9156		
	**HWE test**	$\chi 2$= 0.00, *p* = 0.9372,$f$= − 0.01, CI95% (− 0.32; 0.34)			
rs2287987 T > C	**ERAP1 expression**	**Genotype**	**CC**	**TC**	**TT**
		n	1	16	14
		%	*3.2*	*51.6*	*45.2*
	Non-tumor	Median	0.88		1.16
		CI95%	0.53; 1.26		0.79; 1.54
		Difference	d = − 0.28 CI95% (− 0.81; 0.25), *p* = 0.2848		
	Tumor	Median	0.67		1.08
		CI95%	0.37; 0.98		0.59; 1.69
		Difference	d = − 0.36 CI95% (− 1.00; 0.19), *p* = 0.2224		
	**HWE test**	$\chi 2$=1.98, *p* = 0.1616,$f$= − 0.25, CI95% (− 0.51; 0.06)			
rs30187 C > T	**ERAP1 expression**	**Genotype**	**TT**	**CT**	**CC**
		n	3	16	16
		%	*8.6*	*45.7*	*45.7*
	Non-tumor	Median	1.13		0.83
		CI95%	0.79; 1.47		0.52; 1.16
		Difference	d = 0.30 CI95% (− 0.16; 0.77), *p* = 0.2138		
	Tumor	Median	0.99		0.66
		CI95%	0.60; 1.49		0.35; 0.99
		Difference	d = 0.30 CI95% (− 0.15; 0.82), *p* = 0.2132		
	**HWE test**	$\chi 2$=0.13, *p* = 0.7375,$f$= − 0.06, CI95% (− 0.37; 0.27)			
rs27044 C > G	**ERAP1 expression**	**Genotype**	**GG**	**CG**	**CC**
		n	1	14	19
		%	2.9	41.2	55.9
	Non-tumor	Median	0.92		1.00
		CI95%	0.56; 1.28		0.67; 1.36
		Difference	d = − 0.09 CI95% (− 0.58; 0.42), *p* = 0.7392		
	Tumor	Median	0.95		0.70
		CI95%	0.47; 1.56		0.44; 0.96
		Difference	d = 0.19 CI95% (− 0.30; 0.80), *p* = 0.4736		
	**HWE test**	$\chi 2$=0.71, *p* = 0.3891,$f$= − 0.14, CI95% (− 0.39; 0.14)			

^a^Hodges – Lehmann estimator

^b^Estimated with bootstrap sampling

^c^Distribution for null H0: *locus is in HWE*, estimated exactly with Monte Carlo simulation

^d^Pooled in one group

## Discussion

The results of our previous work [10], presenting *ERAP1* as a susceptibility *locus* for NSCLC, prompted us to conduct further research on this topic. In the present study, we demonstrated a significantly lower level of *ERAP1* mRNA expression in lung cancer tissue compared to adjacent normal tissue. To date, in the literature, there are no reports on *ERAP1* mRNA expression in NSCLC. However, our findings are consistent with the dataset for *ERAP1* mRNA expression in lung adenocarcinoma and lung squamous cell carcinoma deposited in the publicly available database—The Cancer Genome Atlas (TCGA) (http://ualcan.path.uab.edu) [13], (Supplementary Fig. 1). To the best of our knowledge, until now only one study by Fruci et al. [6] investigated ERAP1 expression on the protein level in lung cancer. The authors detected ERAP1 in normal lung bronchial epithelium and myoepithelium, but none of the ten tumor specimens tested were ERAP1 positive. This complete lack of ERAP1 protein in tumors shown by Fruci et al. does not seem to be entirely consistent with our results since we detected *ERAP1* mRNA in almost all tumor tissue samples (albeit in very low levels in some of them). Several possible explanations exist for the observed discrepancy between Fruci et al. and our study. One of them may be related to the regulation of gene expression by microRNA. According to in silico analysis reported in the literature, the *ERAP1* gene can be silenced, among others, by miR-223 [14], and this microRNA was shown to be a validated biomarker for detecting early-stage NSCLC [15]. Nevertheless, further studies are needed to be carried out to find and confirm microRNAs capable of silencing ERAP1 in NSCLC. Another explanation may be the insufficient number of samples tested for the presence of ERAP1 protein in lung cancer tissue included in the study by Fruci et al. [6]. However, it is also worth mentioning that the study by Fruci et al. contradicts the data presented by the Human Protein Atlas [16] on lung cancer (Supplementary Fig. 2). For 6 of the 11 tissues this database showed medium staining (i.e., moderate intensity, > 75% quantity) and only in one tissue ERAP1 was not detected.

Given the limited and inconsistent data on ERAP1 expression in the context of NSCLC, it will be essential to conduct in-depth studies first to determine ERAP1 expression at the protein level, secondly to find out whether there is a correlation between *ERAP1* mRNA and protein expression levels, and finally what mechanisms are responsible for the regulation of ERAP1 protein expression and the reduced mRNA expression observed in the tumor tissue in our study.

Since in the literature there are reports showing an association between some of the *ERAP1* genetic variants and their expression level [17–20], we decided to correlate *ERAP1* mRNA expression levels with the available genotypic data for five *ERAP1* polymorphisms. We showed that carriers of the rs26653C allele (GC + CC) had an almost twofold higher level of *ERAP1* compared to patients with the GG genotype, but the difference was seen only in non-tumor control tissue. Our results are in line with those presented in the GTEx portal [21], indicating rs26653 as an eQTL (expression quantitative trait *locus*) in lung tissue (Supplementary Fig. 3). The rs26653 variant is located in exon 2 and causes a proline to arginine substitution at position 127 of the protein. To date, no report has directly identified rs26653 as a functional variant responsible for altered mRNA expression. Nevertheless, in one study by Mehta et al., heterozygous GC genotype was associated with normal ERAP1 expression in cervical carcinoma as opposed to both homozygotes, which had lower expression (in terms of the proportion of positive cells in immunohistochemistry). Unfortunately, the authors did not provide detailed data documenting this observation [9]. Interestingly, SNP rs26653 was significantly associated with NSCLC in the Chinese population [22], and recently also in Polish Caucasians after adjusting for smoking status [10]. In addition, several previous reports have linked it to some autoimmune diseases, including psoriasis [23–25], ankylosing spondylitis, and inflammatory bowel disease [26]. The association of rs26653 with the disorders mentioned above indicates that it may functionally affect the enzymatic properties of ERAP1, and/or influence gene expression level. Nevertheless, functional studies are needed to examine this supposition. We also cannot entirely exclude the possibility that this non-synonymous SNP may be in strong positive linkage disequilibrium (LD) with the true causal variant. Very recently, a synonymous *ERAP1* variant—rs469783 [Ala637Ala] was also found to be recognized as eQTL. The C allele of this SNP was correlated with higher expression levels of ERAP1 in normal lung tissues and whole blood [20]. Our in silico analyses revealed a weak to moderate LD between this polymorphism and rs26653 (D’ = 0.507, R^2^ = 0.143; CEU population) (https://ldlink.nci.nih.gov/). More interestingly, our further analysis showed that rs26653 is in a moderate to strong LD (D’ = 1.0, R^2^ = 0.637; CEU population) with another synonymous *ERAP1* SNP—rs27434 [Ala356Ala], which according to one study is classified as a disease/trait-associated pQTL (protein quantitative trait *locus*) influencing ERAP1 protein level measured in the cerebrospinal fluid [27]. However, given that the SNPs rs26653 and rs469783 as well as rs27434 are localized in exons, it cannot be ruled out that neither of them is a causative variant controlling ERAP1 expression, which is rather expected to be localized in regulatory elements of the gene, such as the promoter, 5'- and 3'-untranslated regions or even in introns. Further research is necessary to resolve this concern.

It is worth noting that our results suggest that ERAP1 expression in tumor tissue does not seem to be under the direct or indirect control of rs26653, in contrast to what we observed in adjacent normal tissue. Why is that? Similar to many other genes, ERAP1 expression can be down-regulated in cancer cells by different mechanisms, including epigenetics (DNA methylation, histone post-translational modification), transcriptional and translational regulation, as well as post-translational modifications [28, 29]. In lung tumor tissue, the effect of rs26653 (or another SNP being in strong LD with it) on ERAP1 expression may be masked by other mechanisms regulating expression, characteristic of this cancer type.

In our study, the ERAP1 expression level detected in the tumor tissue as well as in the adjacent normal tissue did not affect the overall survival of NSCLC patients. This observation, however, is not consistent with the recent report by Yang et al. [20]. Based on in silico analysis performed with the use of the TCGA database, the authors showed that in non-small cell lung cancer, a higher *ERAP1* mRNA expression level in the tumor correlates with better patient survival. The observed discrepancy may be due to the much smaller number of tested samples included in our analysis, while Yang and coworkers analyzed hundreds of patient datasets deposited in the TCGA database. The second reason may be the length of the observation time of the patients after treatment. While in our study observation time was relatively short (max. 36 months), in the TCGA it was even up to 200 months [20]. A third possibility may be differences in implemented treatment regimens. Both surgery and postoperative treatment are of great importance for overall survival and may significantly mask the impact of the reduced ERAP1 expression. Of note, in our recent work, surgery was the most crucial factor significantly contributing to the extension of the patient's life [10]. Although defects in ERAP1 expression have been detected in various solid tumors, to date the only published report in which lower ERAP1 protein expression in neoplastic tissue was associated with worse survival, as well as the presence of metastases, is cervical carcinoma [9].

Our study revealed significantly lower expression of *ERAP1* mRNA in lung cancer tissue compared to adjacent non-tumor tissue obtained from the same patients. We anticipate that this may be one of the possible mechanisms for avoiding immune recognition. ERAP1 down-regulation may lead to less efficient processing and presentation of tumor antigens, thereby creating a less immunogenic phenotype and facilitating tumor growth and progression. This notion may be supported by findings that in ERAAP-deficient mice (where ERAAP is a mouse homolog of human ERAP1), the generation of some naturally processed peptides in the endoplasmic reticulum was disrupted. In addition, the stability of peptide-MHC-I complexes decreased and CD8 + T cell responses diminished [30]. As Leone et al. [5] point out, “Aberrations in APM genes and proteins have frequently been observed in human tumors and found to correlate with relevant clinical variables, including tumor grade, tumor stage, disease recurrence, and survival. These findings support the idea that APM defects are immune escape mechanisms that disrupt the tumor cells’ ability to be recognized and killed by tumor antigen–specific cytotoxic CD8 + T cells.”

Interestingly, ERAP1 expression varies in different cancers—it can be increased or decreased (the most common situation), but complete loss of expression is not common [5]. This fact may indicate that the complete absence of ERAP1 in the ER might also not be beneficial for cancer development. Presumably, in such a situation, the peptides presented on the surface of tumor cells may be too different from those presented on normal cells, which may stimulate the immune system to fight against the cancer.

A major limitation of the current study is the small number of subjects for whom genotype data were available. Unfortunately, some of our patients were recruited for the need of earlier projects, and we were unable to perform SNP genotyping for them. The second limitation is the lack of data on ERAP1 expression at the protein level; however, we subsequently plan to perform immunohistochemical staining of ERAP1 protein in the same patients and correlate the obtained results with those from the current study.

In conclusion, we showed that *ERAP1* mRNA expression in the non-tumor tissue was significantly higher than in tumor tissue from the same patient. Additionally, we demonstrated that rs26653 could be considered as an expression quantitative trait *locus* associated with ERAP1 expression in normal lung tissue.

## Supplementary Information

**Additional file 1:** **Supplementary Fig. 1.** ERAP1 mRNA expression in normal and primary tumor tissue from patients with Lung Adenocarcinoma (LUAD) (A) and Lung Squamous Cell Carcinoma (LUSC) (B). **Supplemetary Fig. 2.** ERAP1 expression on protein level according to data presented by the Human Protein Atlas for lung cancer. **Supplementary Fig. 3.** GTEx single-tissue eQTLs analysis of association between rs26653G>C and ERAP1 mRNA expression.

## Data Availability

The datasets generated during the current study are available from the corresponding author on reasonable request.
