# Supplementary material for: Down-regulation of ERAP1 mRNA expression in non-small cell lung cancer
Source: BMC Cancer. 2023 Apr 26;23:383. doi: 10.1186/s12885-023-10785-7 (PMC10134604; doi:10.1186/s12885-023-10785-7)
Supplement: Supplementary file 1 — Additional file 1: Supplementary Fig. 1. ERAP1 mRNA expression in normal and primary tumor tissue from patients with Lung Adenocarcinoma (LUAD) (A) and Lung Squamous Cell Carcinoma (LUSC) (B). Supplemetary Fig. 2. ERAP1 expression on protein level according to data presented by the Human Protein Atlas for lung cancer. Supplementary Fig. 3. GTEx single-tissue eQTLs analysis of association between rs26653G>C and ERAP1 mRNA expression. [file 12885_2023_10785_MOESM1_ESM.docx]

**Supplementary Material**

**for publication**

**Down-regulation of ERAP1 mRNA expression in non-small cell lung cancer**

^1^Marta Wagner, ^2^Maciej Sobczyński, ^1^Monika Jasek, ^3^Konrad Pawełczyk, ^4^Irena Porębska, ^5^Piotr Kuśnierczyk, ^5^Andrzej Wiśniewski*

^1^Laboratory of Genetics and Epigenetics of Human Diseases, Hirszfeld Institute of Immunology and Experimental Therapy, Polish Academy of Sciences, Wrocław, Poland; ^2^Laboratory of Molecular Neurobiology, Nencki Institute of Experimental Biology of the Polish Academy of Sciences, Warsaw, Poland; ^3^Department of Thoracic Surgery, Lower Silesian Centre of Oncology, Pulmonology and Haematology, Wrocław, Poland; ^4^Department of Pulmonology and Lung Oncology, Wrocław Medical University, Wrocław, Poland; ^5^Laboratory of Immunogenetics and Tissue Immunology, Hirszfeld Institute of Immunology and Experimental Therapy, Polish Academy of Sciences, Wrocław, Poland

**(A)**

**
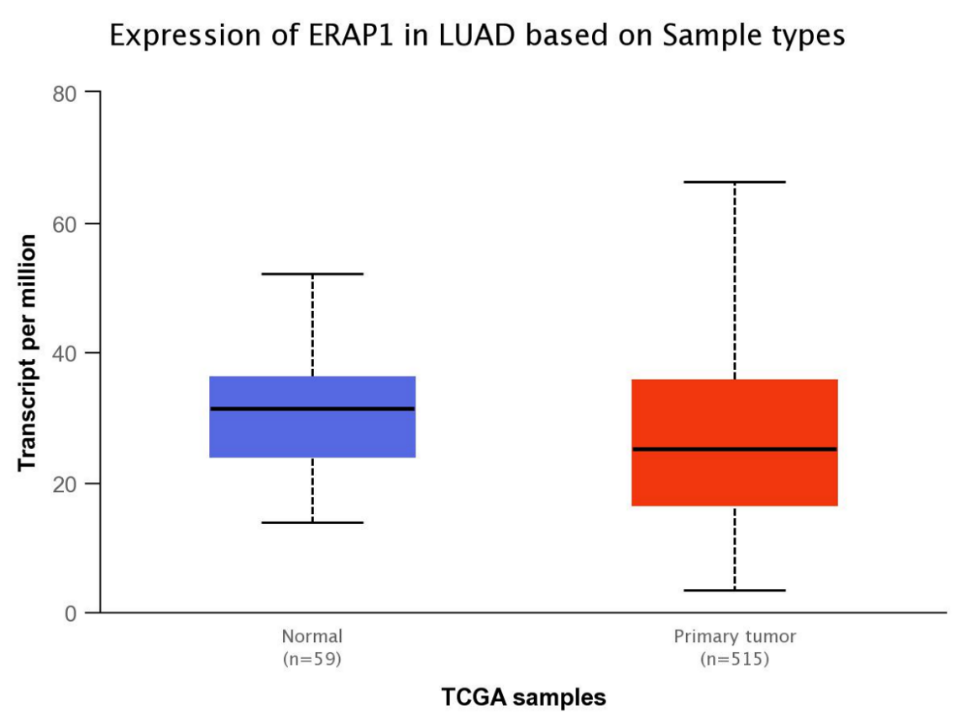
**

**(B)**

**
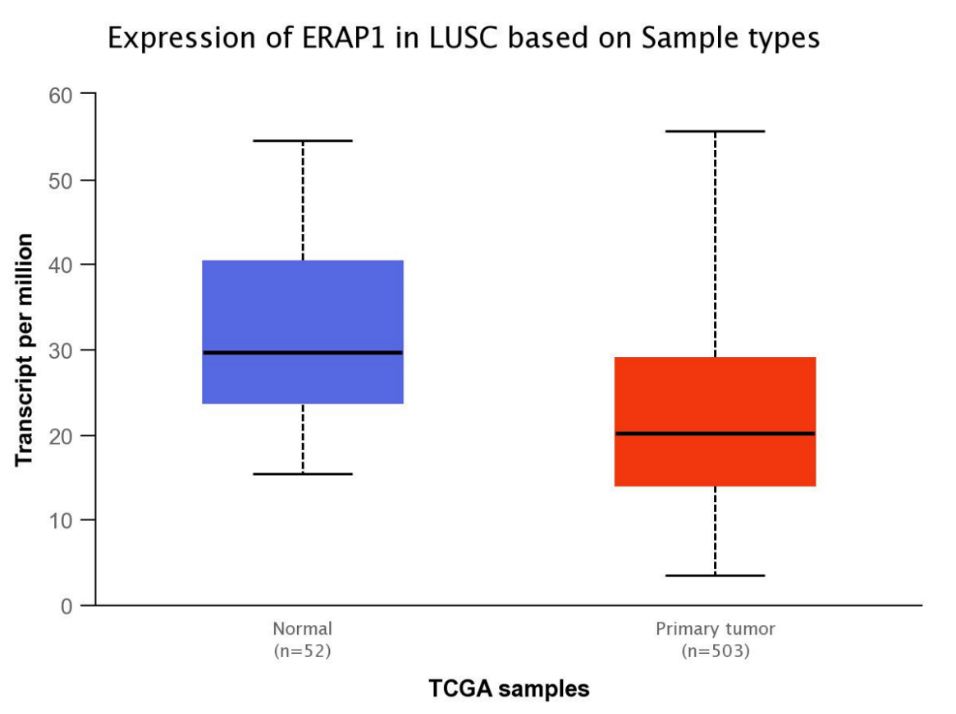
**

**Supplementary Fig. 1** *ERAP1* mRNA expression in normal and primary tumor tissue from patients with Lung Adenocarcinoma (LUAD) **(A)** and Lung Squamous Cell Carcinoma (LUSC) **(B)**


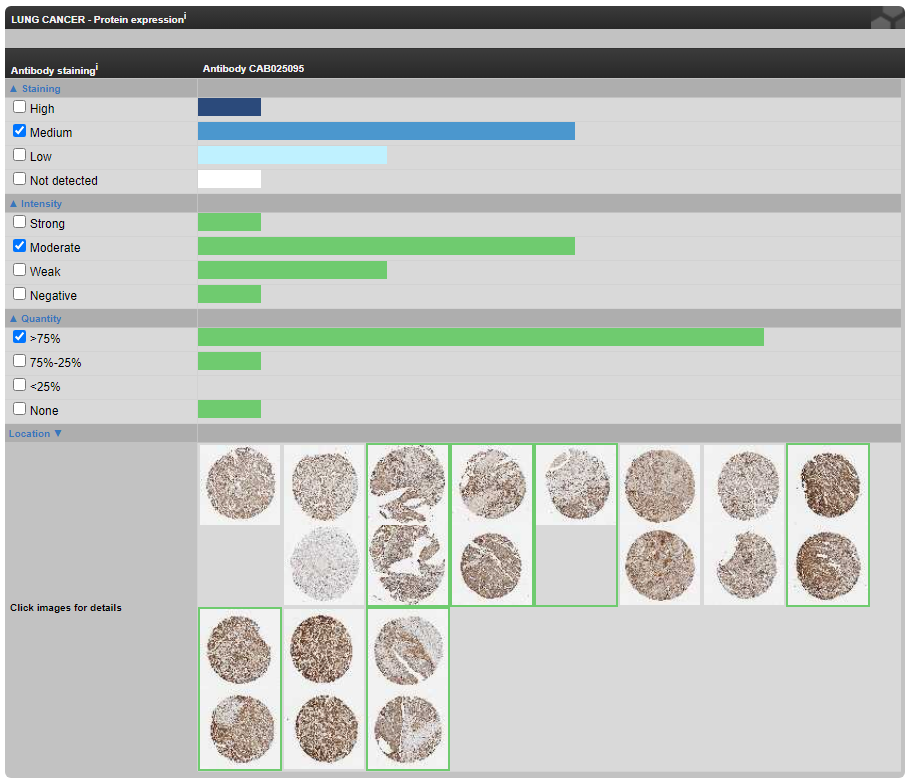


Image from Human Protein Atlas proteinatlas.org;

https://www.proteinatlas.org/ENSG00000164307-ERAP1/pathology

**Supplemetary Fig. 2** ERAP1 expression on protein level according to data presented by the Human Protein Atlas for lung cancer

Of the 11 tissue:

6 - medium staining (i.e., moderate intensity, >75% quantity) depicted in green

1 – high

1 – not detected

3 - low


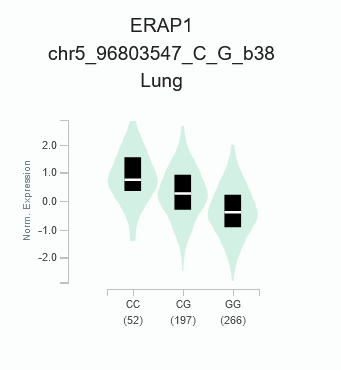


**Supplementary Fig. 3** GTEx single-tissue eQTLs analysis of association between rs26653G>C and *ERAP1* mRNA expression
